# Immediate tumor resection in patients with locally advanced gastroesophageal adenocarcinoma with nonresponse to chemotherapy after 4 weeks of treatment versus resection after completion of chemotherapy (OPTITREAT trial, DRKS00004668): study protocol for a randomized controlled pilot trial

**DOI:** 10.1186/s40814-016-0059-x

**Published:** 2016-04-04

**Authors:** Susanne Blank, Phillip Knebel, Georg-Martin Haag, Thomas Bruckner, Ulla Klaiber, Maria Burian, Anja Schaible, Leila Sisic, Thomas Schmidt, Markus K. Diener, Katja Ott

**Affiliations:** 1 Department of General, Visceral and Transplantation Surgery, University of Heidelberg, Im Neuenheimer Feld 110, Heidelberg, 69120 Germany; 2 Study Centre of the German Surgical Society (SDGC), University of Heidelberg, Im Neuenheimer Feld 110, Heidelberg, 69120 Germany; 3 Department of Medical Oncology, National Center of Tumor Diseases, University Hospital Heidelberg, Im Neuenheimer Feld 460, Heidelberg, 69120 Germany; 4 Institute of Medical Statistics and Informatics, University of Heidelberg, Im Neuenheimer Feld 305, Heidelberg, 69120 Germany; 5 Department of General Visceral and Transplantation Surgery, Endoscopic Center, University of Gießen, Rudolf-Buchheimstr. 7, Gießen, 35392 Germany; 6 Interdisciplinary Endoscopic Center, University of Heidelberg, Im Neuenheimer Feld 460, Heidelberg, 69120 Germany; 7 Department of Surgery, RoMed Klinikum, Pettenkoferstr. 10, Rosenheim, 83022 Germany

**Keywords:** Esophagogastric cancer, Neoadjvuant chemotherapy, Response evaluation

## Abstract

**Background:**

Neoadjuvant chemotherapy is a standard of care for patients with adenocarcinoma of the esophagus and stomach in Europe, but still only 20–40 % respond to therapy and the critical issue; how to treat nonresponding patients is still unclear. So far, there is no randomized trial evaluating the impact of early termination of neoadjuvant chemotherapy and immediate tumor resection in nonresponding patients with locally advanced gastroesophageal cancer on postoperative outcome. With this exploratory pilot trial, we want to get first estimates about the effect of discontinuation of chemotherapy with the aim to plan and conduct a further definitive trial.

**Methods/design:**

OPTITREAT is designed as a single-center, randomized controlled pilot trial with two parallel study groups. Four weeks after starting neoadjuvant chemotherapy in all patients, clinical response will be assessed by endoscopy and endosonographic ultrasound. Then, nonresponding patients (*n* = 84) will be randomized in a 1:1 ratio to intervention group with stopping chemotherapy and immediate tumor resection or control group with completion of chemotherapy before surgery. Outcome measures are overall survival, R0 resection rate, perioperative morbidity and mortality, histopathological response, and quality of life. Statistical analysis will be based on the intention-to-treat population. Due to the study design as an explorative pilot trial, no formal sample size calculation was performed. The planned total sample size of 120 patients is considered ethical and large enough to show the feasibility and safety of the concept. First data on differences between the study groups in the defined endpoints will also be generated.

**Discussion:**

Individualized therapy is of utmost interest in the treatment of locally advanced gastroesophageal adenocarcinoma as less than half of the patients show objective response to current chemotherapy regimens. The findings of the OPTITREAT trial will help to get first data about clinical response evaluation followed by immediate tumor resection in nonresponding patients after 4 weeks of neoadjuvant chemotherapy. Based on the results of this pilot study, a future confirmatory trial will be planned to prove efficacy and evaluate significance.

**Trial registration:**

German Clinical Trial Register number: DRKS00004668

## Background

### Rationale and preliminary data

Based on a recent meta-analysis [1] and three randomized trials [2–4], standard treatment for locally advanced gastroesophageal adenocarcinomas consists of perioperative/neoadjuvant chemotherapy or alternatively preoperative radiochemotherapy for tumors of the gastroesophageal junction. Perioperative chemotherapy demonstrated a survival benefit of about 13 % after 5 years for patients with adenocarcinoma of the esophagus or stomach. However, it is generally accepted that patients with initially locally advanced tumors who respond to chemotherapy have excellent 5-year survival rates of up to 70 % [5] whereas nonresponding patients have significantly worse survival rates of 15–30 % [6, 7]. Some authors even suggest that nonresponding patients could have a worse prognosis [8], lower complete resection rates [9], and an increased morbidity after chemotherapy compared to primarily resected patients. Especially for patients with esophageal cancer, there is evidence that nonresponding patients tend to have a higher perioperative complication rate and a higher mortality [10]. Additionally, only 25–50 % of the patients respond to neoadjuvant chemotherapy [5–7] depending on the applied chemotherapy regimen and the type of response evaluation applied. The need for individualized therapy regimens for patients with gastroesophageal adenocarcinomas is obvious. Until now, it has not been possible to predict response to chemotherapy by molecular markers and to select those patients who benefit from neoadjvuant chemotherapy pretherapeutically [11–18].

Therefore, the evaluation of nonresponding patients at least early during preoperative treatment with the possibility to adapt the further therapy would be of utmost interest. Stopping an ineffective therapy would avoid toxicity, costs, and a decreased quality of life due to secondary effects of the chemotherapy.

### Aims and objective

The primary objective of the OPTITREAT trial is to get first data about early termination of neoadjuvant chemotherapy in nonresponding patients with locally advanced gastroesophageal cancer.

With this exploratory pilot trial, we want to get initial estimates of survival of the study population as well as response rates during chemotherapy for being able to plan a definitive trial. Furthermore, we want to determine whether stopping treatment has any unforeseen detrimental effects on the patients with respect to short- and long-term survival as well as if there are any unforeseen risks by stopping chemotherapy resulting in a higher perioperative morbidity and or mortality. Additionally, we want to get first data about the influence of discontinuation of chemotherapy on quality of life.

## Methods/design

### Study population and eligibility criteria

The study population consists of all patients with histologically proven, resectable gastroesophageal adenocarcinoma who are staged cT2/3/4 and/or cN+ by endoscopy, endoscopic ultrasound, and computerized tomography (CT) scan and thereby meet the criteria and clinical condition for neoadjuvant/perioperative chemotherapy. Patients will be informed about the purpose of the trial, potential benefits, and risks at the Department of General, Visceral and Transplantation Surgery of the University of Heidelberg or at the Department of Medical Oncology at the National Center for Tumor Diseases Heidelberg (NCT). Screening and information will be organized and overviewed by the Clinical Study Center, Heidelberg. If an informed consent is given and all inclusion criteria are fulfilled and exclusion criteria are adhered to (Table 1), patients will be included into the trial. After inclusion, all patients will receive neoadjuvant chemotherapy and 4 weeks after the first day of chemotherapy, patients will undergo endoscopy and endoscopic ultrasound to assess clinical response. Patients who were screened but not enrolled in the trial will be documented in the screening log and the reason for exclusion will be recorded.

**Table 1:** Inclusion and exclusion criteria

Inclusion criteria
Histologically proven adenocarcinoma of the esophagus or stomach
Clinical staging cT3/4 and/or cN+
Resectable primary tumor
Patient considered to tolerate chemotherapy as well as surgery
≥18 years of age
Written informed consent
Exclusion criteria
Lack of understanding or language problems
Expected lack of compliance
Patients not eligible for surgery (ASA ≥ IV)
Distant metastases (M1) without possibility of complete resection (R0)
Neoadjuvant radiochemotherapy

*ASA* American Society of Anaesthesiologists classification

### Sample size

**Fig. 1:**
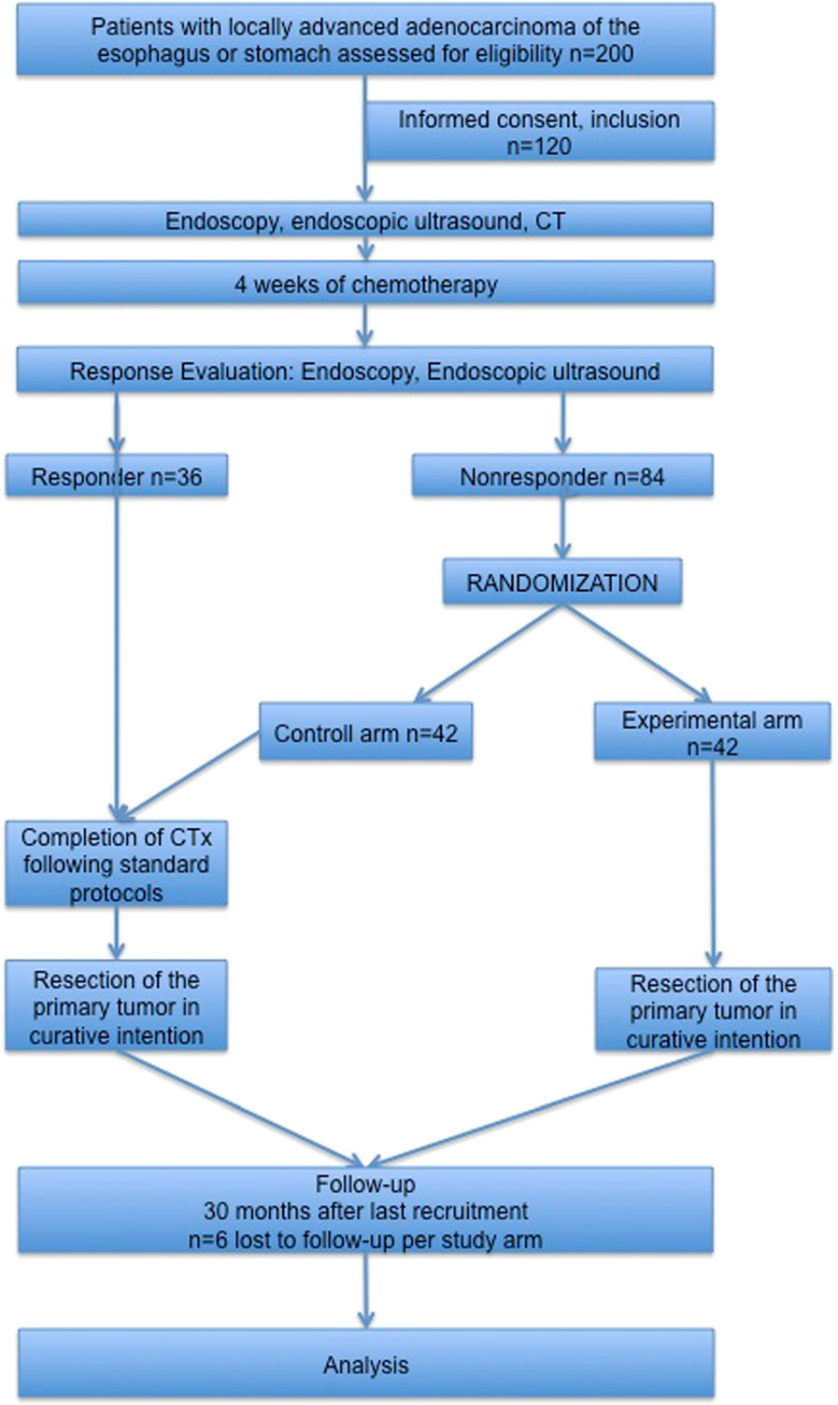
Study Flow Chart

The number of eligible patients will be 120, and among them, the number of patients meeting the criteria for nonresponse is estimated to be 84 assuming a response rate of 30 % [3, 5, 6, 19]. Thus, 42 nonresponding patients per group are estimated to be randomized. To reach that number, a total of 200 patients with locally advanced carcinoma of the esophagus or stomach are estimated to be assessed for eligibility (Fig. 1).

### Trial design

The OPTITREAT trial is designed as a single-center, explorative, randomized controlled pilot trial with two parallel study groups.

### Recruitment and trial timeline

Patient recruitment started in September 2014 and is expected to be as long as 24 months. The follow-up period is 30 months and total duration of the trial is estimated to be 6 years including analysis.

### Randomization

After undergoing clinical response evaluation (see below), all patients defined as nonresponders will be allocated to one of the two treatment arms. In order to achieve comparable study groups, patients will be randomly assigned 1:1 to interventional or control group using block randomization and applying a central online randomization system (randomizer.at).

### Interventions and examinations

All patients will receive neoadjuvant chemotherapy for at least 4 weeks. Neoadjuvant chemotherapy should include a platinum compound and a fluoropyrimidine according to the data from the MAGIC trial [3] and the French ACCORD 07 trial [2]; optionally, a taxane or an anthracycline will be chosen by the oncologist according to local standards and in consideration of patient’s performance status and comorbidities.

Table 2 summarizes the possible chemotherapy schedules.

**Table 2:** Chemotherapy schedules

2-week protocols	
FLOT	FLO
Docetaxel 50 mg/m^2^ 1 h d (day)1	Oxaliplatin 85 mg/m^2^ 2 h d1
Oxaliplatin 85 mg/m^2^ 2 h d1	Folic acid 200 mg/m^2^ 2 h d1
Folic acid 200 mg/m^2^ 2 h d1	5-Fluorouracil 2600 mg/m^2^ 24 h d1 repeat d 15
5-Fluorouracil 2600 mg/m^2^ 24 h d1 repeat d 15	
3-week protocols	
ECF	ECX
Epirubicin 50 mg/m^2^ push d1	Epirubicin 50 mg/m^2^ push d1
Cisplatin 60 mg/m^2^ 2 h d1	Cisplatin 60 mg/m^2^ 2 h d1
5-Fluorouracil 200 mg/m^2^ 24 h d1–21 repeat d 22	Capecitabine 625 mg/m^2^ 2×/d p.o. d1–21 repeat d 22
EOF	EOX
Epirubicin 50 mg/m^2^ push d1	Epirubicin 50 mg/m^2^ push d1
Oxaliplatin 130 mg/m^2^ 2 h d1	Oxaliplatin 130 mg/m^2^ 2 h d1
5-Fluorouracil 200 mg/m^2^ 24 h d1–21 repeat d 22	Capecitabine 625 mg/m^2^ 2×/d p.o. d1–21 repeat d 22

Before starting chemotherapy, endoscopy and endoscopic ultrasound as well as CT scan will be performed to obtain initial clinical tumor staging according to the TNM Classification for Malignant Tumors, 7th edition (2010). Endoscopy and endoscopic ultrasound will be repeated 4 weeks after the beginning of chemotherapy, i.e., on days 12–14 of cycle 2 in case of a 2-week protocol or on days 5–7 of cycle 2 in case of a 3-week protocol. Clinical response will be assessed during the second endoscopy according to defined criteria (Table 3), which have already been shown to be of prognostic relevance [20–22]. Video/digital image documentation will be done to compare the initial diagnostic findings with the following examinations 4 weeks later.

**Table 3:** Criteria for clinical response evaluation [20–22]

	Endoscopy		Endoscopic ultrasound		Others
Responder	Major reduction of tumor size (>75 %) with significant flattening of the tumor	and	More than 50 % reduction of wall thickness (at the largest tumor diameter)	–	–
Nonresponder	Visible residual tumor	and/or	Less than 50 % reduction of wall thickness (at the largest tumor diameter)	and/or	Metastatic lesions

In case of delay of the second chemotherapy cycle, e.g., because of side effects, cytopenia, or other causes, clinical response evaluation will be performed on days 12–14 and days 5–7 of the second cycle, respectively.

In patients with clinical response, neoadjuvant therapy will be completed to a total of 4 cycles in case of a 2-week protocol or to a total of 3 cycles in case of a 3-week protocol. Clinical nonresponders will be randomized to one of the two following study groups:

#### Experimental intervention

Neoadjuvant chemotherapy will be stopped after 4 weeks, and tumor resection will be performed between 1 and 3 weeks after termination of chemotherapy.

#### Control intervention

Neoadjuvant chemotherapy will be completed as intended in the respective chemotherapy regimen, and tumor resection will be performed between 1 and 3 weeks after the end of chemotherapy.

Tumor resection will be done according to the local standards of the center. Surgery will be open access surgery. In patients with adenocarcinoma of the esophagogastric junction (AEG) I/(II), either an Ivor Lewis procedure, i.e., abdominothoracic approach with intrathoracic anastomosis, including a two-field lymphadenectomy or a transhiatal esophagectomy with cervical anastomosis will be performed. Both procedures include an abdominal D-2 lymphadenectomy. Patients with type (II)/III tumors will undergo a transhiatal extended gastrectomy and an extended D2-lymphadenectomy, i.e., resection of the lymph node groups 1 and 2 according to the Japanese Research Society for Gastric Cancer. In patients with tumor localization in the middle or distal third of the stomach and for distal gastric tumors, a total gastrectomy with D2-lymphadenectomy and a subtotal gastrectomy including a D2-lymphadenectomy, if an adequate proximal resection margin is possible, will be performed, respectively.

#### Permitted and not permitted medications/treatments

The administered chemotherapy protocol including dose reduction, stopping, and delay of administration of chemotherapy will be monitored during the whole study. During the postoperative course, adjuvant chemotherapy will not be changed.

Concomitant supportive therapy, e.g., antiemetics, hydration, blood transfusions, and hematopoetic growth factors, will be applied according to the local standards of the center. Toxicities requiring dose adjustment, termination, or switch of the whole protocol or of single chemotherapy agents will be managed according to the choice of the investigator.

Any protocol violation will be documented in the case report form (CRF).

#### Risks

For clinical response evaluation, all patients included in the trial will undergo a second endoscopy and endoscopic ultrasound examination, which are no standard procedures outside the trial. These examinations go along with a potential risk of associated complications, e.g., teeth trauma, bleeding of the mucosal layer, perforation of the esophagus or stomach with possible indication for urgent surgery, postinterventional dysphagia, and allergic reactions as well as complications due to the analgosedation. Patients will be informed about these complications before inclusion as well as at least 24 h before each intervention. Besides this, no additional risks are expected for participants.

### Outcomes and assessment

The following outcome measures will be assessed:

ᅟ *Overall survival*: the time from the day of randomization to the day of death due to any reason. After randomization, patients will be followed up for a minimum duration of 30 months or until death. Patients who have not died by the end of follow-up will be censored at their last contact date, as will be those patients who will be withdrawn from the study for a reason other than death (e.g., lost to follow-up).
ᅟ *Rate of complete tumor resection* (*R0 resection rate*): the frequency of R0 resections as stated in the histology report.^1^
ᅟ *Perioperative morbidity*: the frequency and type of perioperative complications as defined in Table 4 from the day of surgery until 3 months after the index operation.

**Table 4:** Definitions of postoperative morbidity

Anastomotic leakage	Loss of integrity of the anastomosis, confirmed by appearance of contrast medium outside the anastomosis in the abdominal or pleural cavity after oral ingestion of contrast medium or by endoscopy
Leakage of the duodenal stump	Loss of integrity of the duodenal stump leading to diffusion of bile and pancreatic juice to the abdominal cavity
Pancreatic fistula	Drain output of any measurable volume of fluid on or after postoperative day 3 with an amylase content greater than 3 times the serum amylase activity
	Classification (according to the ISGPS definition):
	Grade A: clinically not apparent, well condition, no infectious signs
	Grade B: infectious sign but no sepsis, persistent drainage, no reoperation
	Grade C: sepsis and/or reoperation necessary
Postoperative hemorrhage	Drop of systemic hemoglobin ≥3 g/dl compared to postoperative baseline level and/or need for transfusion of >2 units of packed red blood cells due to intraabdominal hemorrhage as indicated by blood loss via the abdominal drains and/or interventional treatment
Abscess	Closed collection of pus in the abdominal or pleural cavity
Wound healing problems	Leading to the necessity of a special wound care
Lymph fistula	Caused by damage of a lymphatic duct, leading to diffusion of chylus in the abdominal cavity. Diagnosis is done by measurement of triglyceride level in the abdominal drain. A triglyceride level three times higher than serum level is defined as lymphatic fistula.
Chylothorax	Accumulation of chylus in the thoracic cavity caused by damage of the thoracic duct or other intrathoracal lymphatic ducts
Tracheal lesions	Fistulas between esophagus and trachea, as well as loss of integrity of the tracheal wall
Deep vein thrombosis	Formation of a new thrombus in a deep vein, clinically evident (swollen/livid leg, pain), verified by Doppler ultrasound or CT angiography
Pulmonary embolisms	Emboli in the main pulmonary artery or its branches, clinically evident (tachypnea, tachycardia) and verified by CT angiography
Pulmonary infection	At least 3 of 4 of the following:
	Temperature >37.5 °C
	Purulent tracheal secretion
	White blood count >12,000 or <4500/ml
	Elevated CRP level
	As well as radiological evidence of pulmonary infection
Renal failure	Renal failure of sudden onset after operation: doubling of preoperative serum, creatinine level, or need for dialysis or hemofiltration (in patients who were not on dialysis preoperatively)
Cerebral insult	Acute cerebral hypoperfusion, clinically evident by neurological symptoms, verified by cerebral CT scan and/or CT angiography
Myocardial infarction	Clinical symptoms of myocardial infarction as well as heart enzyme (troponin T) changes suggestive of myocardial infarction, changes in electrocardiogram for STEMIs, or evidence of myocardial infarction on coronary angiogram
ᅟ *Perioperative mortality*: the frequency of deaths due to any reason occurring within 30 days after surgery or within the initial hospital stay for resection of the primary tumor.
ᅟ *Clinical response*: as described above and defined in Table 2.
ᅟ *Histopathological response*: the histopathological tumor regression will be assessed using the Becker regression score compromising four tumor regression grades: 1a—no residual tumor, 1b—<10 % viable tumor, 2—10–50 % viable tumor, and 3—>50 % viable tumor [23]. According to Becker’s suggestion, regression scores 1a and 1b both indicate response, whereas regression scores 2 and 3 indicate nonresponse.^1^
ᅟ *Quality of life*: the EORTC QLQ-C30 instrument and the specific esophagogastric module EORTC QLQ-OG25 will be used to assess patients’ quality of life.

#### Study visits

From screening day to day of discharge, there will be five study visits assessing baseline data and demographics, eligibility criteria, endoscopic findings and clinical response, randomization, surgical procedure including resection status and histopathological response, and perioperative morbidity and mortality as well as details of chemotherapy, quality of life, and safety (Table 5). After discharge from hospital, treating oncologists as well as patients will be contacted by phone and in written form to obtain follow-up data every 3 months during the first year after surgery, every 6 months during the second year and once per year afterwards until death or end of study.

**Table 5:** Study visits in the OPTITREAT trial

Documentation	Visit 1	Visit 2	Visit 3	Visit 4	Visit 5	FU	FU	FU	FU	FU	FU	FU
	Screening	Before CTx	4 weeks after CTx	Before surgery	Discharge	3 months	6 months	9 months	12 months	18 months	24 months	30 months
Baseline data, demographics	X											
Eligibility criteria	X											
Endoscopy		X	X									
Clinical response			X									
Randomization			X									
Quality of life	X			X					X			
Information about CTx				X								
R0 resection					X							
Histopathological response					X							
Information about surgery					X							
Perioperative morbidity					X	X						
Perioperative mortalitiy					X	X						
Severe adverse events		X	X	X	X	X						
Survival analysis/recurrence						X	X	X	X	X	X	X

*CTx* chemotherapy, *FU* follow-up

#### Safety evaluations and reporting of adverse events

The incidence of all serious adverse events (SAEs) that occur from the day of inclusion until study termination will be closely monitored and evaluated. A SAE is defined as any adverse event occurring at any time during the period of observation that results in death, is immediately life-threatening, requires, or prolongs hospitalization, results in persistent or significant disability or incapacity, and/or requires medical or other intervention to prevent permanent impairment or damage. All SAEs will be documented on specific SAE forms, and a detailed description of the SAE including the type of event, treatment and seriousness of SAE, causality to trial intervention, outcome, and end of SAE will be given. Postoperative complications which are documented as endpoints in the CRF will not be reported as SAE.

#### Data and safety monitoring board

A data and safety monitoring board (DSMB) composed of independent experts was established before starting the trial. In case of any irregularities, e.g., concerning the frequency or type of reported SAE, the principal investigator will inform the members of the DSMB without delay. Every 20 randomized patients, the DSMB will receive a written safety report. Then, the members of the DSMB will evaluate the benefit/risk ratio and will give appropriate recommendations concerning the continuation of the trial.

### Statistical methods

#### Sample size calculation

Due to the study design as an explorative pilot trial, no formal sample size calculation was performed. A total of 84 nonresponding patients are estimated to be randomized in a 1:1 ratio using block randomization. Since response status of the individual patient cannot be defined at the initial stage of the trial, 120 patients in total will have to be included assuming a response rate of 30 %. The formal sample size is chosen to get a reasonable number of patients in the intended recruitment timeline of 2 years.

#### Statistical analysis

The statistical analysis will be of descriptive nature, the resulting *p* values will not have any confirmative value. Statistical methods will be used to assess the quality of data, homogeneity of treatment groups, endpoints, and safety of the three treatment groups. The analysis will be performed on the basis of an intention to treat (ITT) population and with respect to ITT principles. A patient belongs to the ITT population, when the patient was found to be suitable for the study, signed the informed consent, and finishes the first part of initial chemotherapy. As sensitivity analysis, the endpoints will also be analyzed on the basis of the per protocol (PP) population, where all patients are enclosed, which will finish the trial without protocol deviations

Baseline characteristics, efficacy, and safety endpoints will be analyzed with descriptive methods. The description of continuous variables will include at least the number of observations, mean, 95 % confidence intervals, standard deviation, and median as well as minimum and maximum in the trial population. The description of categorial variables will include at least the number and percentage (including 95 % confidence intervals) of patients belonging to the relevant categories in the trial population as well as in each treatment group.

Possible differences between the two groups of nonresponding patients will be tested for outcome measures using *t* test in case of continuous variables and with chi-square test in case of categorical variables. Possible differences in overall survival will be assessed using Kaplan-Meier method including log-rank test. As stated above, these tests are of descriptive nature without any confirmatory value.

#### Documentation and data handling

The investigator or a designated representative will complete the CRF for the documentation of all protocol-required information. After completing the CRF, it will be reviewed and signed by the investigator and sent to the Institute of Medical Biometrics and Informatics (IMBI), University of Heidelberg. The IMBI will be in charge of the data management within the trial. All data management procedures will be carried out according to the current standard operating procedures (SOPs) of the IMBI.

Clinical monitoring will be performed by the Study Center of the German Surgical Society (SDGC).

#### Withdrawals

Subjects will be free to withdraw their consent at any time without providing a specific reason and without any disadvantage for them. In case of withdrawal, patients will be asked if they agree to be included in the analysis till the point of withdrawal or if they should be taken completely out of the analysis and data should be destroyed.

#### Subject information and informed consent

Patients will be informed about the trial including the procedures, possible hazards to which the individual patient will be exposed as well as the mechanism of treatment allocation in oral and written form. After reading the information form and having been explained the trial including its consequences in an understandable form, the individual patients will have to give written consent to participate in the trial. No interventions or measurements in relation to the trial will be made before.

### Ethical and legal aspects

#### Approval

Prior to the start of the trial, the protocol was presented to the independent ethics committee of the University of Heidelberg and written approval was obtained on 16.07.2013 (reference number: S-637/2012).

#### Good clinical practice

This trial is accomplished in conformity with the principals of the Declaration of Helsinki and the Guidelines for Good Clinical Practice in their current revision. The trial will be carried out in keeping with national and international legal and regulatory requirements.

#### Registration

The trial protocol has been registered with the German Clinical Trials Register (DRKS; DRKS00004668).

## Discussion

Individualized therapy regimens for patients with gastroesophageal adenocarcinomas are more and more requested as response rates and prognosis are not satisfying. Until now, it has not been possible to predict response to chemotherapy by molecular markers and to select those patients who benefit from neoadjvuant chemotherapy pretherapeutically [11–18].

Therapy monitoring with positron emission tomography using F-18fluorodeoxyglucose (FDG-PET) after 2 weeks of chemotherapy has only been evaluated for AEG I and II in clinical trials and cannot be transferred to clinical routine due to a lack of multicentric validation. Additionally, the value of FDG-PET for response evaluation in gastric cancer is clearly limited, since in 50 % of the patients with diffuse gastric cancer tumors do not uptake FGD [20] and data on prognosis are rare and inconclusive [24]. However, the unicentric MUNICON I trial [9] showed for the first time the feasibility of a PET-guided treatment algorithm after only 2 weeks of chemotherapy. In nonresponding patients, chemotherapy was stopped after 2 weeks and these patients were immediately transferred to surgery. None of the patients classified as nonresponders by FDG-PET showed a histopathological response in the resected specimen. The median survival of those patients was 26 months which was better than the median survival of 18 months in a historical control group of nonresponding patients with the completion of chemotherapy within 3 months. Thus, there is evidence that nonresponding patients seem not to be harmed by stopping ineffective chemotherapy. However, these results must be interpreted with caution as data from randomized trials are lacking.

So far, the following methods are available for response evaluation with varying acceptance: clinical response evaluation by endoscopy and CT scan during (extremely rare) or after neoadjuvant chemotherapy [5, 22, 25–27], histopathological regression of the resected specimen [23, 28–30], and early response evaluation by FDG-PET for AEG I and II within a limited number of studies [9, 20, 22, 31, 32]. All the abovementioned methods have shown significant prognostic relevance in various studies [9, 20, 22, 28, 32]. However, the most common methods are clinical and histopathological response evaluation after completion of chemotherapy or resection. As clinical and histopathological response evaluation after completion of therapy or resection cannot be used for therapy stratification, an early clinical response evaluation during treatment is highly warranted.

Response to chemotherapy was first reported to be of utmost importance for prognosis in gastric cancer in 1999 by Lowy et al. who considered both clinical and histopathological response evaluation [5]. Response was either defined clinically by the reduction of more than 50 % in endoscopy, gastrointestinal series, and CT scan or less than 10 % of residual tumor in the resected specimen. Since then, the prognostic relevance for histopathological regression and clinical response has been confirmed in several studies [6, 19, 28, 30, 33, 34].

Clinical response, defined as nearly total flattening in endoscopy and more than 50 % reduction in size measured by endoluminal ultrasound (identical criterion as in this study) or CT scan, showed prognostic relevance in patients with AEG I/II with a median survival of >53 months in case of response and 13 months in case of nonreponse (*p* = 0.01) [22]. In gastric cancer, endoscopic response evaluation after 50 % of the planned dose of chemotherapy has been shown to correlate well with the final histopathological response evaluation and prognosis in a limited number of patients. All included patients received 2 cycles of chemotherapy. After the first cycle of chemotherapy, the negative predictive value for response evaluation by endoscopy was 93.9 % and the positive predictive value was 41.7 % [35]. Consequently, the identification of nonresponding patients seems reliable in a high percentage. Therefore and considering the possible harms of the continuation of a probably ineffective chemotherapy, the discontinuation of chemotherapy should not be disadvantageous for patients included in this trial. As data from a recent study indicate that the length of chemotherapy does neither influence response, complete resection rate, nor prognosis of the patients [36], premature termination of neoadjuvant chemotherapy in nonresponding patients might be considerably beneficial for patients regarding prognosis, costs, and quality of life.

Based on the abovementioned data, a clinical response evaluation after 4 weeks of preoperative chemotherapy by endoscopy and endoscopic ultrasound seems to be appropriate to tailor treatment of the nonresponding patients. Furthermore, as evidence from well-designed studies is lacking and the critical issue, how to treat nonresponding patients, is still unclear, clinical equipoise is given within this trial.

A randomized trial is warranted and needed to evaluate the impact of immediate tumor resection in patients with locally advanced gastroesophageal adenocarcinoma with nonresponse to chemotherapy to resection after completion of chemotherapy. A future definitive trial would be a noninferiority trial aimed at demonstrating that stopping chemotherapy early and operating sooner would not lead to a worse outcome. When assuming event rates of 0.3 in both groups and assuming a noninferiority margin for the hazard ratio of 0.8, a total sample size of 2100 patients would be necessary, with power of 0.8 and a level of significance of 0.05 (calculated with Addplan 6.0).

The findings of this current study will give first information about clinical response evaluation by endoscopy and endoscopic ultrasound followed by discontinuation of neoadjuvant chemotherapy in nonresponding patients. Based on the results, the possibility to conduct a future confirmatory trial of high quality will be evaluated with the aim to prove efficacy and evaluate significance of this method. Data from this current exploratory pilot trial is essential in revising the assumptions made in sample size calculation to determine the required sample size for a definitive trial.

### Trial status

Patient recruitment began in September 2014, and recruiting of patients is still ongoing.

## Endnote

^1^Pathology records are assessed by a pathological team specialized on upper gastrointenstinal tumors.
